# Vascular changes of the choroid and their correlations with visual acuity in diabetic retinopathy

**DOI:** 10.3389/fendo.2024.1327325

**Published:** 2024-02-23

**Authors:** Ruixia Jing, Xiubin Sun, Jimin Cheng, Xue Li, Zhen Wang

**Affiliations:** ^1^ Jinan Central Hospital, Shandong First Medical University & Shandong Academy of Medical Sciences, Jinan, China; ^2^ Department of Biostatistics, School of Public Health, Cheeloo Collage of Medicine, Shandong University, Jinan, China; ^3^ Department of Ophthalmology, Central Hospital Affiliated to Shandong First Medical University, Jinan, China

**Keywords:** diabetes mellitus, diabetic retinopathy, optical coherence tomography, choroidal vascular index, visual acuity

## Abstract

**Objective:**

To investigate changes in the choroidal vasculature and their correlations with visual acuity in diabetic retinopathy (DR).

**Methods:**

The cohort was composed of 225 eyes from 225 subjects, including 60 eyes from 60 subjects with healthy control, 55 eyes from 55 subjects without DR, 46 eyes from 46 subjects with nonproliferative diabetic retinopathy (NPDR), 21 eyes from 21 subjects with proliferative diabetic retinopathy (PDR), and 43 eyes from 43 subjects with clinically significant macular edema (CSME). Swept-source optical coherence tomography (SS-OCT) was used to image the eyes with a 12-mm radial line scan protocol. The parameters for 6-mm diameters of region centered on the macular fovea were analyzed. Initially, a custom deep learning algorithm based on a modified residual U-Net architecture was utilized for choroidal boundary segmentation. Subsequently, the SS-OCT image was binarized and the Niblack-based automatic local threshold algorithm was employed to calibrate subfoveal choroidal thickness (SFCT), luminal area (LA), and stromal area (SA) by determining the distance between the two boundaries. Finally, the ratio of LA and total choroidal area (SA + LA) was defined as the choroidal vascularity index (CVI). The choroidal parameters in five groups were compared, and correlations of the choroidal parameters with age, gender, duration of diabetes mellitus (DM), glycated hemoglobin (HbA1c), fasting blood sugar, SFCT and best-corrected visual acuity (BCVA) were analyzed.

**Results:**

The CVI, SFCT, LA, and SA values of patients with DR were found to be significantly lower compared to both healthy patients and patients without DR (*P* < 0.05). The SFCT was significantly higher in NPDR group compared to the No DR group (*P* < 0.001). Additionally, the SFCT was lower in the PDR group compared to the NPDR group (*P* = 0.014). Furthermore, there was a gradual decrease in CVI with progression of diabetic retinopathy, reaching its lowest value in the PDR group. However, the CVI of the CSME group exhibited a marginally closer proximity to that of the NPDR group. The multivariate regression analysis revealed a positive correlation between CVI and the duration of DM as well as LA (*P* < 0.05). The results of both univariate and multivariate regression analyses demonstrated a significant positive correlation between CVI and BCVA (*P* = 0.003).

**Conclusion:**

Choroidal vascular alterations, especially decreased CVI, occurred in patients with DR. The CVI decreased with duration of DM and was correlated with visual impairment, indicating that the CVI might be a reliable imaging biomarker to monitor the progression of DR.

## 1 Introduction

By 2045, it is expected that the number of adults worldwide with diabetes mellitus (DM) will exceed 700 million (1). Diabetic retinopathy (DR), which is the most prevalent microvascular complication of DM, remains a leading cause of blindness among the working population (2, 3). It has been reported that approximately one-third of DM patients suffer from DR (4).

The deterioration of vision caused by DR is a prevalent risk factor for permanent blindness in patients with DM (5). Nonproliferative diabetic retinopathy (NPDR) is characterized by asymptomatic microvascular changes or retinal microleakage, which can eventually progress to proliferative diabetic retinopathy (PDR) or diabetic macular edema (DME) as DM advances (6, 7). Therefore, the importance of early diagnosis of DR and the study of its risk factors cannot be ignored.

There was a certain relationship between DR and choroidal thickness (CT) (8). Research conducted by Wang et al. (9) revealed that CT plays a crucial role in the development of DR. Optical coherence tomography (OCT), widely recognized as a valuable non-invasive imaging technique, can be utilized for choroid imaging (10). OCT-derived CT has been proposed as a quantitative index for evaluating choroidal structure and function. Several studies conducted in the past decade have analyzed changes in CT thickness in patients with DR, but the results obtained vary. Some reports showed thinning of CT, while others show no change or even thickening. However, these studies were limited to investigating only CT (11–15). Recently, other choroidal vascular parameters such as the choroidal vascular index (CVI) and the choroidal luminal area (LA) have been reported in age-related macular degeneration (AMD), pathological myopia (PM), and other diseases related to the choroid (16–19). There were also studies that showed a relationship between CVI and the progression of DR, but all of them had limitations. Kim et al. only focused on studying CVI in the 1,500 μm area of the fovea, while Gupta et al. utilized spectral domain optical coherence tomography (SD-OCT) (20, 21).

In this study, we employed the residual U-Net deep learning algorithm, which has been previously reported, to automatically segment and quantify CT and choroidal vasculature in swept source optical coherence tomography (SS-OCT) images (16, 22, 23). We analyzed the correlation between choroidal vascular changes and DR to provide new insights into the prevention and treatment of DR.

Headings: CVI correlations with visual acuity in DR.

## 2 Methods

### 2.1 Study design

This was a retrospective study approved by the Ethics Review Committee of the Central Hospital affiliated with Shandong First Medical University, which followed the basic principles of the Declaration of Helsinki. As this was a retrospective study, written informed consent was waived by the Ethics Review Board. All analytical data has been anonymized.

### 2.2 Patients

This study included a total of 165 patients (165 eyes) with type 2 DM and 60 healthy patients (60 eyes) who underwent SS-OCT examination at the Department of Ophthalmology, Central Hospital Affiliated to Shandong First Medical University from September 2022 to September 2023. The diagnosis of type 2 DM was based on the criteria set by the American Diabetes Association (24).

Inclusion criteria: Age >18 years. Exclusion criteria: (1) Refractive error > +3.0 D or refractive error < - 3.0 D (spherical equivalent (SE)); (2) Hypertension; (3) Ocular trauma; (4) Retinal laser photocoagulation; (5) Intraocular surgery; (6) Intravitreal injection; (7) Presence of other retinal diseases, such as AMD, retinal arteriovenous occlusion, or neurodegenerative disease; (8) Glaucoma; (9) Hypertrophic choroidal pigment epitheliopathy, such as central serous chorioretinopathy; (10) History of inflammation; (11) Refractive media opacification affecting fundus examination.

### 2.3 Ophthalmologic examination

Demographic information, comprehensive medical history, and ophthalmic history were recorded during the initial visit. All patients underwent a comprehensive eye examination, which included measurements of Snellen best-corrected visual acuity (BCVA), intraocular pressure (IOP), autorefraction, slit lamp biomicroscopy, dilated fundus examination, fundus photography, and SS-OCT.

### 2.4 Acquiring and analyzing OCT images

The SS-OCT (VG200S; SVision Imaging, Henan, China) device with a central wavelength of 1050 nm was used for OCT imaging. The scanning speed was 100,000 A-scans/s. The axial optical resolution was 3.8 μm, and the axial digital resolution was 2.0 μm. The maximum scanning depth of the posterior segment was 9 mm. To obtain two transverse lines passing through the fovea, a radial line scan measuring 12 mm in length was performed. In order to thoroughly examine changes in choroidal vascular around the macula, we analyzed cross-sectional images within a radius of 6 mm centered on the fovea. The examinations were conducted by an experienced ophthalmic technologist, with a minimum signal strength requirement of 4 as recommended by the manufacture.

Before conducting image analysis, we used Bennett’s formula (*t = p × q × s*) to correct the OCT image and eliminate the influence of axial length (AL) on image size, based on previous studies (25–27). Here, *t* represents the actual scan length, *p* denotes the magnification factor of the OCT imaging system, *q* refers to the eye-related magnification factor, and *s* represents the raw measurement value of the OCT imaging system.

**Figure 1:**
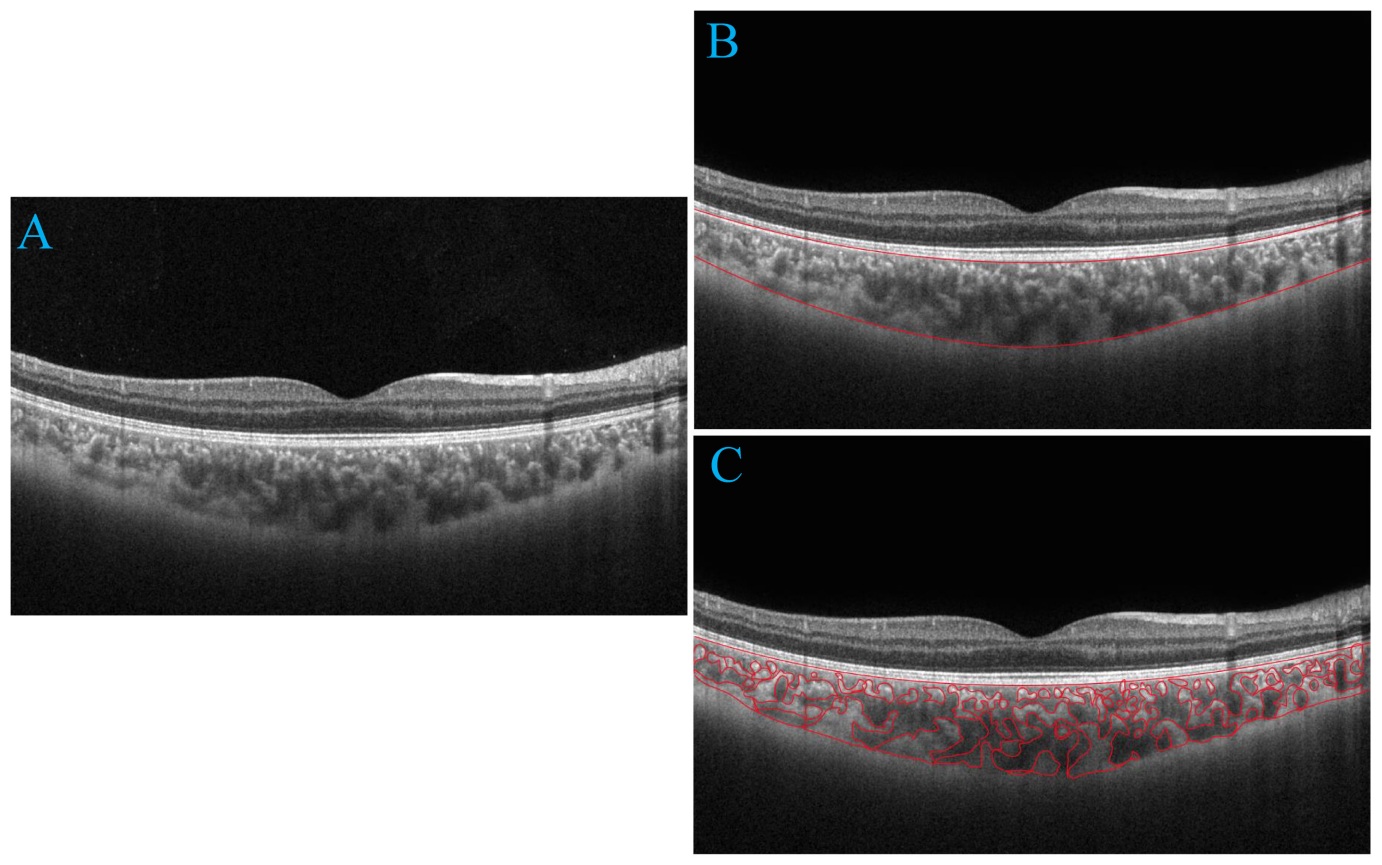
Progression of choroidal image segmentation. The original SS-OCT image **(A)**. Segmentation of the choroidal area **(B)**. Overlay of the region of interest created after performing image binarization on the SS-OCT image **(C)**.

We utilized deep learning techniques to automatically quantify choroidal parameters within a circular region with a diameter of 6.0 mm, using the macular fovea as the central point. The choroidal boundary was automatically segmented in all images using deep learning algorithms based on the modified residual U-Net method. Residual U-Net, a residual unit used as a replacement for the traditional convolution unit, was employed in deep networks. It has been observed that residual U-Net can achieve better performance with fewer parameters compared to traditional methods. The thickness between the retinal pigment epithelium (RPE) layer and the junction of the choroid and sclera is defined as CT. After binarizing the OCT images, we applied Niblack’s automatic local threshold algorithm to calibrate both the LA and stromal area (SA). The choroidal vascular index (CVI) was defined as the ratio of LA to the total choroidal area (SA + LA) (**Figure 1**).

### 2.5 Basic information and laboratory tests

The study collected data from medical records, including factors such as age, gender, duration of DM, glycated hemoglobin (HbA1c), fasting blood sugar, and body mass index (BMI). Age was defined as the age at the first visit. The duration of DM was defined as the time from the diagnosis of DM to the first eye examination. Fasting blood sugar and HbA1c were recorded as the most recent results in the case system, and BMI was calculated by dividing weight (kg) by height squared (m).

### 2.6 Patient screening and grouping

Healthy patients were defined as controls. The DM patients were initially classified into four groups (No DR, NPDR, PDR, and CSME) by two retinal specialists independently (R. J. and X. L.) based on the International DR Staging Criteria (28). In case of disagreement between the two retinal specialists, a more senior retinal specialist (Z. W.) made the final decision. One eye of each patient was randomly selected for analysis during the study.

### 2.7 Statistical analysis

The Statistical Package for the Social Sciences (version 26.0, IBM SPSS) was used for data analysis. When appropriate, study data were reported as mean ± standard deviation (SD), median (interquartile range, IQR), frequencies, or percentages. The Shapiro-Wilk test was employed to assess whether quantitative variables followed a normal distribution. Data that adhered to a normal distribution were described using mean ± SD. Data that exhibited a skewed distribution were presented as median (IQR). Fisher’s exact test was utilized to analyze categorical variables. For normally distributed data, one-way analysis of variance (ANOVA) was applied. The Kruskal-Wallis test was used for analyzing skewed data. Univariate and multivariate linear regression analyses were conducted to investigate the factors influencing choroidal characteristics. Results with a *P* value less than 0.05 were considered statistically significant.

## 3 Results

### 3.1 Basic patient characteristics

A total of 225 eyes were included in this study, which were divided into 5 groups: healthy controls (group 1, n=60), No DR (group 2, n=55), NPDR (group 3, n=46), PDR (group 4, n=21), and CSME (group 5, n=43). The demographic, ocular, and systemic characteristics of the patients were presented in **Table 1**. A total of 165 patients with type 2 DM were included in this study, including 101 females and 64 males, with a mean age of 56.67 ± 6.22 years. The mean duration of DM was 9.16 ± 4.03 years. There were no significant differences in age and gender within the study group. DM duration (*P* < 0.001), BMI (*P* = 0.034), HbA1c (*P* < 0.001), and fasting blood sugar (*P* < 0.001) showed significant differences between each DR group. BCVA logMAR was significantly lower in CSME compared to the other study groups (*P* < 0.001). BCVA logMAR did not differ between No DR and NPDR (*P* = 0.041). BCVA logMAR *showed* slight difference between NPDR and PDR (*P* = 0.728). Systolic blood pressure, IOP, and SE did not show significant differences among the study groups (*P* > 0.05).

**Table 1:** Demographic and clinical characteristics of the eyes (n = 225).

Variables	Healthy controls (n = 60)	No DR (n = 55)	NPDR (n = 46)	PDR (n = 21)	CSME (n = 43)	*P* value
No. of eyes	60	55	46	21	43	
Age, years, median (IQR)	54.50 (46-69)	56 (46-49)	55 (47-69)	54 (47-69)	56 (47-69)	0.204^*^
Sex, n (%)						0.347^ɫ^
Female	36	31	29	14	27	
Male	24	24	17	7	16	
BMI (Kg/m^2^), median (IQR)	21.55 (19.5-24.6)	22.4 (19.8-24.9)	23.1 (19.8-34.0)	23.0 (19.7-24.6)	23 (19.6-25.6)	< 0.001^*^
DM duration, year, median (IQR)		5 (1-12)	10 (7-15)	15 (9-18)	11 (6-15)	< 0.001^*^
HbA1c, %, median (IQR)		6.1 (5.2-6.8)	7 (6.5-7.5)	7.8 (6.8-8.8)	7.2 (6.0-8.0)	< 0.001^*^
Fasting blood sugar, mmol/L, median (IQR)		7.5 (7.0-9.1)	7.9 (7.0-9.0)	8.6 (7.6-9.0)	8.2 (7.1-10.2)	< 0.001^*^
Systolic BP, mmHg, median (IQR)		117 (105-138)	115.5 (106-135)	123 (107-129)	118 (108-127)	0.595^*^
Diastolic BP, mmHg, median (IQR)		68 (62-76)	65.5 (60-76)	65 (63-72)	64 (60-71)	**< 0.001** ^*^
RAAS inhibitors, use, n, %		10.9%	17.39%	52.38%	34.88%	**<0.001^ɫ^**
Ophthalmologic examination						
BCVA, logMAR, median (IQR)	0.1 (0-0.3)	0.1 (0-0.4)	0.1 (0-0.4)	0.2 (0-0.5)	0.6 (0.4-0.8)	< 0.001^*^
SE, diopter, median (IQR)	0.50 (-1.50-1.50)	0 (-1.50-1.50)	0 (-1.50-1.00)	0 (-1.25-1.25)	0.50 (-1.25-1.50)	0.066^*^
IOP, mmHg, median (IQR)	14 (12-17)	14 (12-17)	14 (13-17)	14 (12-17)	14 (12-17)	0.195^*^

DR, diabetic retinopathy; NPDR, nonproliferative diabetic retinopathy; PDR, proliferative diabetic retinopathy; CSME, clinically significant macular edema; IQR, interquartile range; BMI, body mass index; DM, diabetes mellitus; HbA1c, glycated hemoglobin; BP, blood pressure; RAAS, renin-angiotensin-aldosterone system; BCVA, best-corrected visual acuity; logMAR, logarithm of the minimum angle of resolution; SE, spherical equivalent. IOP, intraocular pressure; *Kruskal-Wallis test; ^ɫ^Fisher exact text; P with statistical significance is shown in boldface.

### 3.2 Subfoveal choroidal thickness and CVI

**Figure 2:**
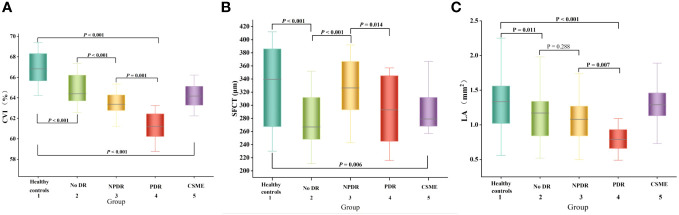
Box-and-whisker plots comparing the choroidal parameters at the vertical meridian among the five groups: **(A)** CVI; **(B)** SFCT; **(C)** LA. The median is represented by the middle line within each box, and the second and third quartiles are represented by the lower and upper segments of the box, respectively.

The SFCT measurement results of each group were shown in **Figure 2B**. The SFCT values for groups 1-5 were 331.67 ± 58.43μm, 278.36 ± 41.48μm, 326.57 ± 45.12μm, 291.29 ± 48.94μm, and 297.23 ± 42.40μm, respectively. The thickness of SFCT in the eyes of patients with DM was found to be thinner compared to that in healthy controls (*P* < 0.05). Notably, the SFCT in the No DR group was significantly lower than both the healthy controls group (*P* < 0.001) and the NPDR group (*P* < 0.001). Additionally, the SFCT in the PDR group was significantly lower than that in the NPDR group (*P* = 0.014). These findings indicated that SFCT was significantly thinner in the eyes of DM patients and that PDR patients have even thinner SFCT compared to other DR patients.

In the univariate regression analyses, there were no significant associations observed between gender, duration of DM, fasting blood sugar, HbA1c, systolic blood pressure, renin-angiotensin-aldosterone system (RAAS) inhibitor use, spherical equivalent (SE), or intraocular pressure (IOP) and SFCT (*P* > 0.05) (**Table 2**). However, age showed a significant correlation with SFCT (*P* = 0.032), along with BMI showing a significant correlation as well (*P* = 0.007), and diastolic blood pressure demonstrating a highly significant association with SFCT (*P* < 0.001) (**Table 2**). Nevertheless, in the multivariate regression analysis only diastolic blood pressure remained significantly correlated with SFCT (*P* = 0.001) (**Table 2**).

**Table 2:** Linear regression analyses of factors associated with SFCT.

Variables	Univariate		Multivariate	
	Standardized β	P value	Standardized β	*P* value
Age, years	-0.143	**0.032**	-0.110	0.171
Sex, n (%)	-0.043	-0.521	-	-
Female				
Male				
BMI (Kg/m^2^)	-0.179	**0.007**	-0.121	0.137
DM duration, year	0.018	0.821	-	-
HbA1c, %	0.029	0.701	-	-
Fasting blood sugar, mmol/L	0.052	0.508	-	-
Systolic BP, mmHg	0.026	0.742	-	-
Diastolic BP, mmHg	0.290	**< 0.001**	-3.306	**0.001**
RAAS inhibitors, use, n, %	-0.032	0.679	-	-
Ophthalmologic examination				
SE, diopter	-1.09	0.102	-	-
IOP, mmHg	-0.071	0.292	-	-

SFCT, Subfoveal choroidal thickness; BMI, body mass index; DM, diabetes mellitus; HbA1c, glycated hemoglobin; BP, blood pressure; RAAS, renin-angiotensin-aldosterone system; SE, spherical equivalent. IOP, intraocular pressure; P with statistical significance is shown in boldface.

The CVI measurement results of each group are presented in **Figure 2A**. The CVI values for groups 1-5 were as follows: 66.93 ± 1.14%, 64.85 ± 1.33%, 63.53 ± 1.04%, 61.25 ± 1.32%, and 64.23 ± 1.04%. Notably, the eyes of DM patients exhibited significantly lower CVI compared to healthy controls (*P* < 0.001). Furthermore, the PDR group demonstrated a significantly lower CVI than both the healthy controls group (*P <*0.001) and NPDR group (*P* = 0.001). These findings indicate a significant reduction in CVI among DM patients, with even lower levels observed in PDR patients.

In the univariate linear regression analysis, gender, age, systolic blood pressure, diastolic blood pressure, SE, IOP, and SA did not show significant correlation with CVI (*P* > 0.05). However, BMI, duration of DM, HbA1c, fasting blood sugar, use of RAAS inhibitors, SFCT, LA, and CVI were found to be significantly correlated (*P* < 0.05) according to **Table 3**. The multivariate linear regression analysis revealed that DM duration and LA remained significantly correlated with CVI after adjusting for other variables (*P* < 0.05) as shown in **Table 3**.

**Table 3:** Linear regression analyses of factors associated with CVI.

Variables	Univariate		Multivariate	
	Standardized β	P value	Standardized β	*P* value
Age, years	0.022	0.735	-	-
Sex, n (%)	0.038	0.573	-	-
Female				
Male				
BMI (Kg/m^2^)	-0.278	**< 0.001**	-0.03	0.650
DM duration, year	-0.426	**< 0.001**	-0.212	**0.016**
HbA1c, %	-0.391	**< 0.001**	-0.041	0.646
Fasting blood sugar, mmol/L	-0.244	**0.002**	-0.036	0.576
Systolic BP, mmHg	-0.046	0.554	-	-
Diastolic BP, mmHg	0.051	0.512	-	-
RAAS inhibitors, use, n, %	0.147	**0.025**	-0.081	0.197
Ophthalmologic examination				
SE, diopter	0.094	0.160	-	-
IOP, mmHg	0.019	0.775	-	-
SFCT, μm	0.154	**0.021**	-0.080	0.221
SA (mm^2^)	-0.003	0.968	-	-
LA (mm^2^)	0.25	**< 0.001**	-1.428	**< 0.001**

CVI, choroidal vascular index; BMI, body mass index; DM, diabetes mellitus; HbA1c, glycated hemoglobin; BP, blood pressure; SFCT, subfoveal choroidal thickness; RAAS, renin-angiotensin-aldosterone system; SE, spherical equivalent. IOP, intraocular pressure; SA, stromal area; LA, luminal area; P with statistical significance is shown in boldface.

The LA measurement results of each group are presented in **Figure 2C**. Notably, the PDR group exhibited a significantly lower LA compared to the other DR groups, suggesting an inverse relationship between LA and CVI.

### 3.3 BCVA logMAR

**Figure 3:**
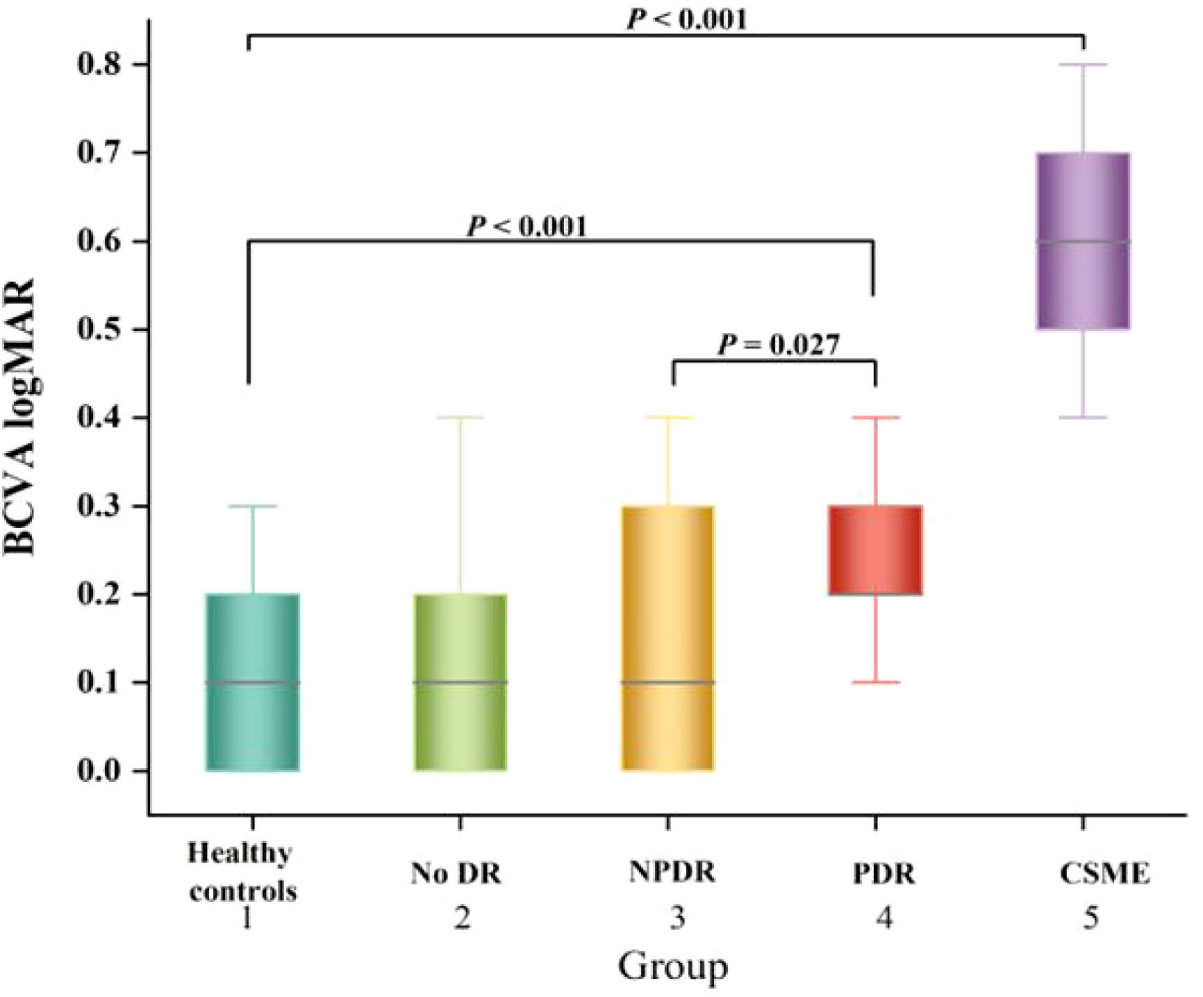
Box-and-whisker plots comparing the BCVA logMAR at the vertical meridian among the five groups. The median is represented by the middle line within each box, and the second and third quartiles are represented by the lower and upper segments of the box, respectively.

The BCVA logMAR measurement results of each group were presented in **Figure 3**. The BCVA logMAR for groups 1-5 were as follows: 0.107 ± 0.107, 0.145 ± 0.126, 0.160 ± 0.134, 0.257 ± 0.133, and 0.593 ± 0.118 respectively. The BCVA logMAR of PDR patients was significantly worse compared to that of healthy controls (*P* < 0.001).

The univariate linear regression analysis revealed a significant association between worse BCVA logMAR and several factors in patients with DR, including older age (*P* = 0.006), longer duration of DM (*P* < 0.001), higher levels of HbA1c (*P* = 0.001), elevated fasting blood sugar levels (*P* = 0.002), thinner SFCT (*P* < 0.001), and reduced choroidal vascularity index values, as shown in **Table 4**. Eventually, multivariate linear regression analysis revealed significant correlations between BCVA logMAR and age (*P* = 0.011), duration of DM (*P* = 0.012), fasting blood sugar (*P* = 0.011), and CVI (*P* = 0.003) in patients with DR (**Table 4**).

**Table 4:** Linear regression models based on BCVA.

Variables	Univariate		Multivariate	
	Standardized β	P value	Standardized β	*P* value
Age, years	0.182	**0.006**	0.188	**0.011**
Sex, n (%)	0.005	0.08	-	-
Female				
Male				
DM duration, year	0.333	**< 0.001**	0.255	**0.012**
HbA1c, %	0.262	**0.001**	0.153	0.117
Fasting blood sugar, mmol/L	0.241	**0.002**	0.190	**0.011**
SFCT, μm	-0.246	**< 0.001**	-0.116	0.101
LA (mm^2^)	0.083	0.231	-	**-**
CVI (%)	0.199	**0.003**	0.239	**0.003**

BCVA; best-corrected visual acuity; DM, diabetes mellitus; HbA1c, glycated hemoglobin; SFCT, subfoveal choroidal thickness; LA, luminal area; CVI, choroidal vascular index; P with statistical significance is shown in boldface.

## 4 Discussion

In the present study, SS-OCT was used to evaluate the choroidal structural changes in eyes with DR. The thinning of the choroid found was consistent with previous findings (20, 29). Alterations of the choroidal vasculature in patients with DR was further quantified. We investigated the alterations in choroidal vascular parameters, including CVI, SA, and LA, in both healthy people and patients with DM. In patients with DM, we observed a significant decrease in CVI, LA, and SA in the presence of DR. However, no correlation was found between these parameters and CSME. Furthermore, CVI showed a strong negative association with BCVA, suggesting that patients with lower CVI exhibited poorer visual acuity, which was consistent with previous research findings (30). Consequently, CVI may serve as a reliable biomarker for monitoring the progression of DR; thus, preserving CVI could emerge as a novel clinical objective.

Importantly, the SFCT was found to be thinner in the group without DR compared to healthy controls (*P* < 0.001). However, it was thicker in the NPDR group compared to both the without DR and PDR groups (*P* = 0.014). This suggests that the SFCT initially increases in the early stages of DR and then decreases as the disease progresses. These findings were consistent with the study conducted by Wang et al. (19). The CVI exhibited a gradual decline with the progression of DR (*P* < 0.05). Nevertheless, multivariate linear regression analysis revealed no significant difference between SFCT and CVI, which contrasted with the findings reported by Kim et al. (20). One possible reason for the discrepancy in research results was the difference in the proximity of the CVI studied. While our study focused on CVI within 6 mm of the fovea, their study examined CVI within 1,500 μm of the fovea. Additionally, they grouped patients with NPDR, whereas we did not. This difference in grouping may also had an impact on the research outcomes.

The influence of HbA1c on CT was still in discussion, which has not been adjusted in most previous CT studies. Unsal et al. (31) and Kim et al. (32) found a significant correlation between HbA1c and CT, indicating that HbA1c may be a confounding parameter. This study found no correlation between HbA1c and SFCT, which is consistent with the findings of Wang et al. (33). However, our study revealed a significant association between age and BMI with CT. Shao et al. (34) reported that SFCT was significantly associated with BCVA after adjusting for age, sex, AL, and corneal curvature in the normal population. This study further demonstrated that thicker CT was independently associated with better BCVA in DR patients. Therefore, the CT may be a biomarker for visual function in DR patients.

In this study, we observed a significant impact of HbA1c on CVI (*P* < 0.001). However, after adjusting for age, gender, duration of DM, fasting blood glucose, and using multivariate regression analyses, we discovered that there was no significant correlation between HbA1c and CVI. This finding was inconsistent with the results of Temel E et al. (35), who reported a negative correlation between Hbac1 and CVI. The reason for this difference may be that they did not consider factors such as age, gender, duration of DM.

Previous studies have reported significantly lower CVI in patients with DM and DR (20, 21, 36). However, Kim et al. (20) and Tan et al. (36) did not specifically analyze the changes of CVI in patients with DME. Our study analyzed the changes in CVI in patients with DME as did the study by Gupta et al. (21). Both studies observed a statistically significant decrease in CVI among the DME group when compared to healthy controls. However, our results revealed a higher CVI in the DME group compared to the NPDR and PDR groups, which was not analyzed in their study. Cao et al. (37) found that the loss of choroidal capillaries in the eyes of patients with DM was four times greater than that in nondiabetic patients. The reduction of the choroidal capillary layer (resulting in a decrease in CVI) may contribute to hypoxia in the RPE and outer retinal layer, leading to an upregulation of vascular endothelial growth factor (38). This could also contribute to the deterioration of vision in DR.

In the present study, we found a significant association between worsening BCVA and CVI in DR patients. Multivariate regression analyses revealed that a decrease in CVI was the most relevant factor for visual impairment. Previous studies have not examined the relationship between BCVA and CVI (20, 21). The choroid primarily supplied nutrients and oxygen to the photoreceptors (39). Previous studies have indicated that CT influenced visual impairment caused by photoreceptor degeneration (25). However, our findings showed no significant correlation between BCVA and CT, which was inconsistent with their findings. One possible reason for this discrepancy was that their subjects were patients with PM, who were younger in age, whereas we studied patients with type 2 DM, who were older. In the future, prospective studies with large sample sizes will be needed to investigate whether visual impairment in patients with type 2 DM is influenced by CT.

In this study, CVI was found to be correlated with BCVA, and multivariate regression analyses revealed that a decrease in CVI was the factor most strongly associated with visual impairment. Therefore, we hypothesize that monitoring changes in CVI may provide a reliable parameter for predicting future loss of visual acuity and could serve as a novel target for intervention aimed at preventing the progression of visual impairment in DR.

There were some limitations to this study. Firstly, choroidal parameters were only measured within a 6 mm radius around the macular fovea, but studying larger zones may provide more representative information about CVI. Secondly, some studies have reported diurnal variations in CT obtained by OCT, however, the diurnal variations of choroidal vascular are currently unknown, which may have influenced our results. Thirdly, we included only 21 patients with PDR because we specifically focused on pre-treatment PDR patients, which might have influenced our results. Fourthly, our study of NPDR was not conducted in a graded manner, which may have also influenced our results. Finally, it was important to note that this study was a small retrospective analysis with a limited sample size. The smaller sample size in this study may have implications on the statistical significance of the analysis results, potentially leading to insufficient power. Furthermore, the generalizability of the findings might be constrained due to the limited sample size. Moreover, a smaller sample size can introduce bias and hinder adequate control for potential confounding factors, thereby impacting the accuracy and reliability of the results. The aforementioned limitations need to be addressed in future research.

## 5 Conclusions

In conclusion, this study demonstrated that patients with DM, even without DR, exhibited significantly lower CVI compared to healthy controls. Furthermore, alterations in choroidal structure were found to be significantly associated with visual impairment in DR. CVI can be used as a quantitative parameter to evaluate choroidal damage in patients with DR. CVI may serve as a reliable quantitative biomarker for monitoring the progression of DR. Clinicians should closely monitor changes in choroidal vasculature in patients with DR and recognize that preventing a decline in CVI may be an important clinical goal for preventing the progression of DR.

## Data availability statement

The original contributions presented in the study are included in the article/supplementary materials. Further inquiries can be directed to the corresponding author.

## Ethics statement

The studies involving humans were approved by the Ethics Review Committee of the Central Hospital Affiliated to Shandong First Medical University. The studies were conducted in accordance with the local legislation and institutional requirements. The participants provided their written informed consent to participate in this study.

## Author contributions

RJ: Writing – original draft, Writing – review & editing, Investigation, Software. XS: Methodology, Writing – original draft. JC: Methodology, Writing – original draft. XL: Data curation, Methodology, Writing – original draft. ZW: Writing – review & editing.

## References

[1] Zhang R Dong L Yang Q Liu Y Li H Zhou W. Prophylactic interventions for preventing macular edema after cataract surgery in patients with diabetes: a bayesian network meta-analysis of randomized controlled trials. EClinicalMedicine. (2022) 49:101463. doi: 10.1016/j.eclinm.2022.101463 35747191 PMC9124709

[2] Cheung N Mitchell P Wong TY. Diabetic retinopathy. Lancet. (2010) 376:124–36. doi: 10.1016/S0140-6736(09)62124-3 20580421

[3] Wang W Lo ACY. Diabetic retinopathy: pathophysiology and treatments. Int J Mol Sci. (2018) 19:1816. doi: 10.3390/ijms19061816 29925789 PMC6032159

[4] Wong TY Cheung CM Larsen M Sharma S Simó R. Diabetic retinopathy. Nat Rev Dis Primers. (2016) 2:16012. doi: 10.1038/nrdp.2016.12 27159554

[5] Liu T Lin W Shi G Wang W Feng M Xie X. Retinal and choroidal vascular perfusion and thickness measurement in diabetic retinopathy patients by the swept-source optical coherence tomography angiography. Front Med (Lausanne). (2022) 9:786708. doi: 10.3389/fmed.2022.786708 35372401 PMC8971655

[6] Preethi S Rajalakshmi AR. Proliferative diabetic retinopathy in typical retinitis pigmentosa. BMJ Case Rep. (2015) 2015:bcr2014208589. doi: 10.1136/bcr-2014-208589 PMC4458599 26021380

[7] Shah KB Han DP. Proliferative diabetic retinopathy. Int Ophthalmol Clin. (2004) 44:69–84. doi: 10.1097/00004397-200404440-00007 15577565

[8] Hidayat AA Fine BS. Diabetic choroidopathy. Light and electron microscopic observations of seven cases. Ophthalmology. (1985) 92:512–22. doi: 10.1016/S0161-6420(85)34013-7. 2582331

[9] Wang W Li L Wang J Chen Y Kun X Gong X. Macular choroidal thickness and the risk of referable diabetic retinopathy in type 2 diabetes: a 2-year longitudinal study. Invest Ophthalmol Vis Sci. (2022) 63:9. doi: 10.1167/iovs.63.4.9 PMC9034727 35420642

[10] Cuenca N Ortuño-Lizarán I Sánchez-Sáez X Kutsyr O Albertos-Arranz H Fernández-Sánchez L. Interpretation of OCT and OCTA images from a histological approach: clinical and experimental implications. Prog Retin Eye Res. (2020) 77:100828. doi: 10.1016/j.preteyeres.2019.100828 31911236

[11] Laíns I Talcott KE Santos AR Marques JH Gil P Gil J. Choroidal thickness in diabetic retinopathy assessed with swept-source optical coherence tomography. Retina. (2018) 38:173–82. doi: 10.1097/IAE.0000000000001516 28196053

[12] Abadia B Suñen I Calvo P Bartol F Verdes G Ferreras A. Choroidal thickness measured using swept-source optical coherence tomography is reduced in patients with type 2 diabetes. PloS One. (2018) 13:e0191977. doi: 10.1371/journal.pone.0191977 29394291 PMC5796707

[13] Ambiya V Kumar A Baranwal VK Kapoor G Arora A Kalra N. Change in subfoveal choroidal thickness in diabetes and in various grades of diabetic retinopathy. Int J Retina Vitreous. (2018) 4:34. doi: 10.1186/s40942-018-0136-9 30214825 PMC6134708

[14] Tavares Ferreira J Vicente A Proença R Santos BO Cunha JP Alves M. Choroidal thickness in diabetic patients without diabetic retinopathy. Retina. (2018) 38:795–804. doi: 10.1097/IAE.0000000000001582 28267113

[15] Ohara Z Tabuchi H Nakakura S Yoshizumi Y Sumino H Maeda Y. Changes in choroidal thickness in patients with diabetic retinopathy. Int Ophthalmol. (2018) 38:279–86. doi: 10.1007/s10792-017-0459-9 PMC5876415 28194551

[16] Wang Y Chen S Lin J Chen W Huang H Fan X. Vascular changes of the choroid and their correlations with visual acuity in pathological myopia. Invest Ophthalmol Vis Sci. (2022) 63:20. doi: 10.1167/iovs.63.12.20 PMC9672896 36378132

[17] Koh LHL Agrawal R Khandelwal N Sai Charan L Chhablani J. Choroidal vascular changes in age-related macular degeneration. Acta Ophthalmol. (2017) 95:e597–601. doi: 10.1111/aos.13399 28391615

[18] Rasheed MA Goud A Mohamed A Vupparaboina KK Chhablani J. Change in choroidal vascularity in acute central serous chorioretinopathy. Indian J Ophthalmol. (2018) 66:530–4. doi: 10.4103/ijo.IJO_1160_17 PMC5892056 29582814

[19] Kim RY Chung DH Kim M Park YH. Use of choroidal vascularity index for choroidal structural evaluation in central serous chorioretinopathy with choroidal neovascularization. Retina. (2020) 40:1395–402. doi: 10.1097/IAE.0000000000002585 31259812

[20] Kim M Ha MJ Choi SY Park YH. Choroidal vascularity index in type-2 diabetes analyzed by swept-source optical coherence tomography. Sci Rep. (2018) 8:70. doi: 10.1038/s41598-017-18511-7 29311618 PMC5758605

[21] Gupta C Tan R Mishra C Khandelwal N Raman R Kim R. Choroidal structural analysis in eyes with diabetic retinopathy and diabetic macular edema-A novel OCT based imaging biomarker. PloS One. (2018) 13:e0207435. doi: 10.1371/journal.pone.0207435 30533048 PMC6289408

[22] Zhang H Yang J Zhou K Li F Hu Y Zhao Y. Automatic Segmentation and visualization of choroid in OCT with knowledge infused deep learning. IEEE J BioMed Health Inform. (2020) 24:3408–20. doi: 10.1109/JBHI.2020.3023144 32931435

[23] Zheng G Jiang Y Shi C Miao H. Deep learning algorithms to segment and quantify the choroidal thickness and vasculature in swept-source optical coherence tomography images. J Innov Opt Health Sci. (2021) 14:2140002. doi: 10.1142/S1793545821400022

[24] American Diabetes Association. 2. Classification and diagnosis of diabetes: standards of medical care in diabetes-2018. Diabetes Care. (2018) Suppl 1):S13–27. doi: 10.2337/dc18-S002 29222373

[25] Ye J Shen M Huang S Fan Y Yao A Pan C. Visual acuity in pathological myopia is correlated with the photoreceptor myoid and ellipsoid zone thickness and affected by choroid thickness. Invest Ophthalmol Vis Sci. (2019) 60:1714–23. doi: 10.1167/iovs.18-26086 31013344

[26] Wang Y Ye J Shen M Yao A Xue A Fan Y. Photoreceptor degeneration is correlated with the deterioration of macular retinal sensitivity in high myopia. Invest Ophthalmol Vis Sci. (2019) 60:2800–10. doi: 10.1167/iovs.18-26085 31266057

[27] Yang Y Wang J Jiang H Yang X Feng L Hu L. Retinal microvasculature alteration in high myopia. Invest Ophthalmol Vis Sci. (2016) 57:6020–30. doi: 10.1167/iovs.16-19542 27820633

[28] Wilkinson CP Ferris FL 3rd Klein RE Lee PP Agardh CD Davis M. Proposed international clinical diabetic retinopathy and diabetic macular edema disease severity scales. Ophthalmology. (2003) 110:1677–82. doi: 10.1016/S0161-6420(03)00475-5 13129861

[29] Abadia B Bartol-Puyal FA Calvo P Verdes G Isanta C Pablo LE. Mapping choroidal thickness in patients with type 2 diabetes. Can J Ophthalmol. (2020) 55:45–51. doi: 10.1016/j.jcjo.2019.06.009 31712028

[30] Marques JH Marta A Castro C Baptista PM José D Almeida D. Choroidal changes and associations with visual acuity in diabetic patients. Int J Retina Vitreous. (2022) 8:6. doi: 10.1186/s40942-021-00355-z 34998439 PMC8742927

[31] Unsal E Eltutar K Zirtiloğlu S Dinçer N Ozdoğan Erkul S Güngel H. Choroidal thickness in patients with diabetic retinopathy. Clin Ophthalmol. (2014) 8:637–42. doi: 10.2147/OPTH.S59395 PMC3971934 24707168

[32] Kim JT Lee DH Joe SG Kim JG Yoon YH. Changes in choroidal thickness in relation to the severity of retinopathy and macular edema in type 2 diabetic patients. Invest Ophthalmol Vis Sci. (2013) 54:3378–84. doi: 10.1167/iovs.12-11503 23611988

[33] Wang W Liu S Qiu Z He M Wang L Li Y. Choroidal thickness in diabetes and diabetic retinopathy: a swept source OCT study. Invest Ophthalmol Vis Sci. (2020) 61:29. doi: 10.1167/iovs.61.4.29 PMC7401852 32324858

[34] Shao L Xu L Wei WB Chen CX Du KF Li XP. Visual acuity and subfoveal choroidal thickness: the Beijing Eye Study. Am J Ophthalmol. (2014) 158:702–709.e1. doi: 10.1016/j.ajo.2014.05.023 24878308

[35] Temel E Özcan G Yanık Ö Demirel S Batıoğlu F Kar İ. Choroidal structural alterations in diabetic patients in association with disease duration, HbA1c level, and presence of retinopathy. Int Ophthalmol. (2022) 42:3661–72. doi: 10.1007/s10792-022-02363-w 35604622

[36] Tan KA Laude A Yip V Loo E Wong EP Agrawal R. Choroidal vascularity index - a novel optical coherence tomography parameter for disease monitoring in diabetes mellitus? Acta Ophthalmol. (2016) 94:e612–6. doi: 10.1111/aos.13044 27151819

[37] Cao J McLeod S Merges CA Lutty GA. Choriocapillaris degeneration and related pathologic changes in human diabetic eyes. Arch Ophthalmol. (1998) 116:589–97. doi: 10.1001/archopht.116.5.589 9596494

[38] Shima DT Adamis AP Ferrara N Yeo KT Yeo TK Allende R. Hypoxic induction of endothelial cell growth factors in retinal cells: identification and characterization of vascular endothelial growth factor (VEGF) as the mitogen. Mol Med. (1995) 1:182–93. doi: 10.1007/BF03401566. PMC2229943 8529097

[39] Nickla DL Wallman J. The multifunctional choroid. Prog Retin Eye Res. (2010) 29:144–68. doi: 10.1016/j.preteyeres.2009.12.002 PMC2913695 20044062

